# An overview of advanced biocompatible and biomimetic materials for creation of replacement structures in the musculoskeletal systems: focusing on cartilage tissue engineering

**DOI:** 10.1186/s13036-019-0209-9

**Published:** 2019-11-13

**Authors:** Azizeh Rahmani Del Bakhshayesh, Nahideh Asadi, Alireza Alihemmati, Hamid Tayefi Nasrabadi, Azadeh Montaseri, Soodabeh Davaran, Sepideh Saghati, Abolfazl Akbarzadeh, Ali Abedelahi

**Affiliations:** 1 Drug Applied Research Center, Tabriz University of Medical Sciences, Tabriz, Iran; 2 Department of Tissue Engineering, Faculty of Advanced Medical Sciences, Tabriz University of Medical Sciences, Tabriz, Iran; 3 Student Research Committee, Tabriz University of Medical Sciences, Tabriz, Iran; 4 Department of Nanotechnology, Faculty of Advanced Medical Sciences, Tabriz University of Medical Sciences, Tabriz, Iran

**Keywords:** Cartilage tissue engineering, Biomaterials, Musculoskeletal tissue engineering, Biomimetic materials, Scaffolds, Tissue engineering

## Abstract

Tissue engineering, as an interdisciplinary approach, is seeking to create tissues with optimal performance for clinical applications. Various factors, including cells, biomaterials, cell or tissue culture conditions and signaling molecules such as growth factors, play a vital role in the engineering of tissues. In vivo microenvironment of cells imposes complex and specific stimuli on the cells, and has a direct effect on cellular behavior, including proliferation, differentiation and extracellular matrix (ECM) assembly. Therefore, to create appropriate tissues, the conditions of the natural environment around the cells should be well imitated. Therefore, researchers are trying to develop biomimetic scaffolds that can produce appropriate cellular responses. To achieve this, we need to know enough about biomimetic materials. Scaffolds made of biomaterials in musculoskeletal tissue engineering should also be multifunctional in order to be able to function better in mechanical properties, cell signaling and cell adhesion. Multiple combinations of different biomaterials are used to improve above-mentioned properties of various biomaterials and to better imitate the natural features of musculoskeletal tissue in the culture medium. These improvements ultimately lead to the creation of replacement structures in the musculoskeletal system, which are closer to natural tissues in terms of appearance and function. The present review article is focused on biocompatible and biomimetic materials, which are used in musculoskeletal tissue engineering, in particular, cartilage tissue engineering.

## Introduction

The musculoskeletal system contains a variety of supporting tissues, including muscle, bone, ligament, cartilage, tendon, and meniscus, which support the shape and structure of the body. After severe injuries due to various causes such as severe crashes, diseases, or malignancies (prolonged denervation or aggressive tumor ablation), the lost tissue needs repair or replacement with healthy tissue [1]. Tissue transplantation from a local or remote location is the primary treatment of these problems, which itself causes significant complications [2]. The main problem is the morbidity of the donor’s places caused by loss of function and volume deficiency following the donation. The base of tissue engineering is the imitation of organogenesis that has achieved success in recent years [3]. Engineered biomaterials, as 3-dimensional (3D) structures (scaffolds), have an essential role in the regeneration of the musculoskeletal system. Depending on the type of damaged tissue (cartilage, bone, skeletal muscle, tendon and ligament), an extensive range of natural and non-natural biomaterials as a particular scaffold can be used in this regard [4].

For example, an appropriate scaffold in cartilage tissue engineering should have properties, including appropriate physicochemical properties, simulation of native cartilage ECM, stimulation of cartilage differentiation, biocompatibility, filling of defective areas and adhesion to surrounding tissue. Among the various structures, injectable hydrogels because their properties are essential for cartilage tissue engineering. The hydrated 3D environment of hydrogels can mimic the native ECM of cartilage, can be useful in transporting of nutrients and cellular metabolites and can load and deliver bioactive agents such as drugs and growth factors to target places of cartilage in a minimally invasive way [5]. Also, the porosity of scaffold has a significant role in cartilage tissue engineering. In scaffolds with closed pores, distribution of cells into the scaffold can be limited and lead to the creation of heterogeneous ECM that has poor mechanical properties [6]. Also, in situ forming hydrogels due to their features such as similarity to native ECM and ease implantation by a needle are widely used in bone tissue engineering. Gel-based scaffolds with similar chemical and structural properties to native bone can improve the behavior of stem cells towards bone formation. To have structure with an appropriate osteoconductivity and excellent mechanical properties, incorporation of inorganic materials to hydrogels is promising [7]. The porosity of the scaffold is also significant in bone tissue engineering. Previous studies have shown that the porosity of scaffolds should be more than 80%. Even, pores in the range of between 100 and 500 μm are suitable in this regard. In recent years, hydrogel composite structures have been widely used for bone tissue engineering. The use of glass-ceramics (GC) and bioactive glass (BG) has been considered due to its biomechanical properties, biocompatibility and improved bone tissue formation. GCs and BGs as mineralization factors, which have osteoconductive properties, can support the osteoblast cells. Also, BGs due to their Na, Ca, Si, and P ions can encourage new bone formation in vivo from the osteoblast cells. In some studies, fibrous BG has been used because of its mimicking the ECM [8].

Another component of the musculoskeletal system, which connects muscle to bone, is the tendon that contains densely packed aligned collagen fibers. Therefore, electrospun aligned Nano and micro-fibers can mimic the native tendon tissue in terms of structural and mechanical properties [9]. On the other hand, the base membrane of muscle is mainly composed of laminin and collagen with a tubular structure that supports muscle integrity. The functional muscle tissue is made of fibers covered by basement membrane and is highly aligned and arranged in muscle bundles. In this regard, there are various methods for fabrication of two-dimensional (2D) micro-patterned surfaces such as electrospinning, groove/ridge micro- and Nano-patterns through photolithography or spin coating [10]. Although 2D micro-patterned surfaces can produce align muscle myoblasts and myotubes, the resulting cell sheets have some drawbacks, for example, limited thickness, which makes it difficult to harvest the cell sheets [11]. Therefore, other scaffolds such as three-dimensional (3D) micro-patterned scaffolds have been considered in skeletal muscle tissue engineering. These types of scaffolds can be fabricated by liquid dispensing and freeze-drying. Prepared muscle tissue in 3D micro-patterned scaffolds can be used as a direct implant for tissue repair [12].

In skeletal muscle tissue engineering, scaffolds should be made of electroactive biomaterials to emulate ECM of muscle cells [13]. Various conductive materials such as polypyrrole, polyaniline, and multiwall carbon nanotubes (MWNTs) in combination with polymers have been studied for promoting myogenic differentiation [14]. But, there are some limitations for long-term applications of these materials due to the problems like toxicity, biocompatibility, non-biodegradability, and difficulty in fabricating of 3D scaffold [15, 16]. Moreover, the engineering of muscle tissue appears to be difficult due to its structural complexity. The two main challenges in this regard are the organization of the 3D myotubes in highly aligned structures and the stimulation of the myotubes maturation in terms of improvement of sarcomere [17]. In the previous studies, it has indicated that electrical stimulation can enhance the maturation of myoblasts [18, 19]. But, this approach has some limitation such as process scalability. Also, the role of scaffold stiffness on the elongation, spreading, and the cooperative fusion of myoblasts has been studied [20]. In these studies, it has been indicated that the scaffold stiffness affects the making of syncytia, myotube maturation, and assembling of the sarcomeric unit [21]. According to extensive studies conducted in this regard, it has been shown that various organic and inorganic materials are used in musculoskeletal tissue engineering. This review article discusses the types of different biomaterials used in musculoskeletal tissue engineering either alone or in combination with other materials as scaffolds.

### Biomimetic biomaterials for musculoskeletal tissue engineering

Biomimetic biomaterials are materials that can be employed in biomedical fields, especially in tissue engineering and drug delivery systems. These are used as an implantable device or part of it that protect the damaged tissues of the body or promote tissue formation [22]. In the past, inert materials were considered as ideal materials for medical applications such as metallic materials in orthopedics and silicone for gel breast implants [23]. But since these materials have no interactions with the environment (tissues or fluids), today the attitude of the ideal biomaterial has changed. In particular, the advent of degradable biomaterials has led to advances in new research fields, including tissue engineering and drug delivery [24]. Typically degradable polymers are known as biodegradable biomaterials, and the first usable biodegradable biomaterials are polyesters, which, as a result of degradation, are converted into smaller portions (lactic acid and glycolic acid) [25].

**Fig. 1:**
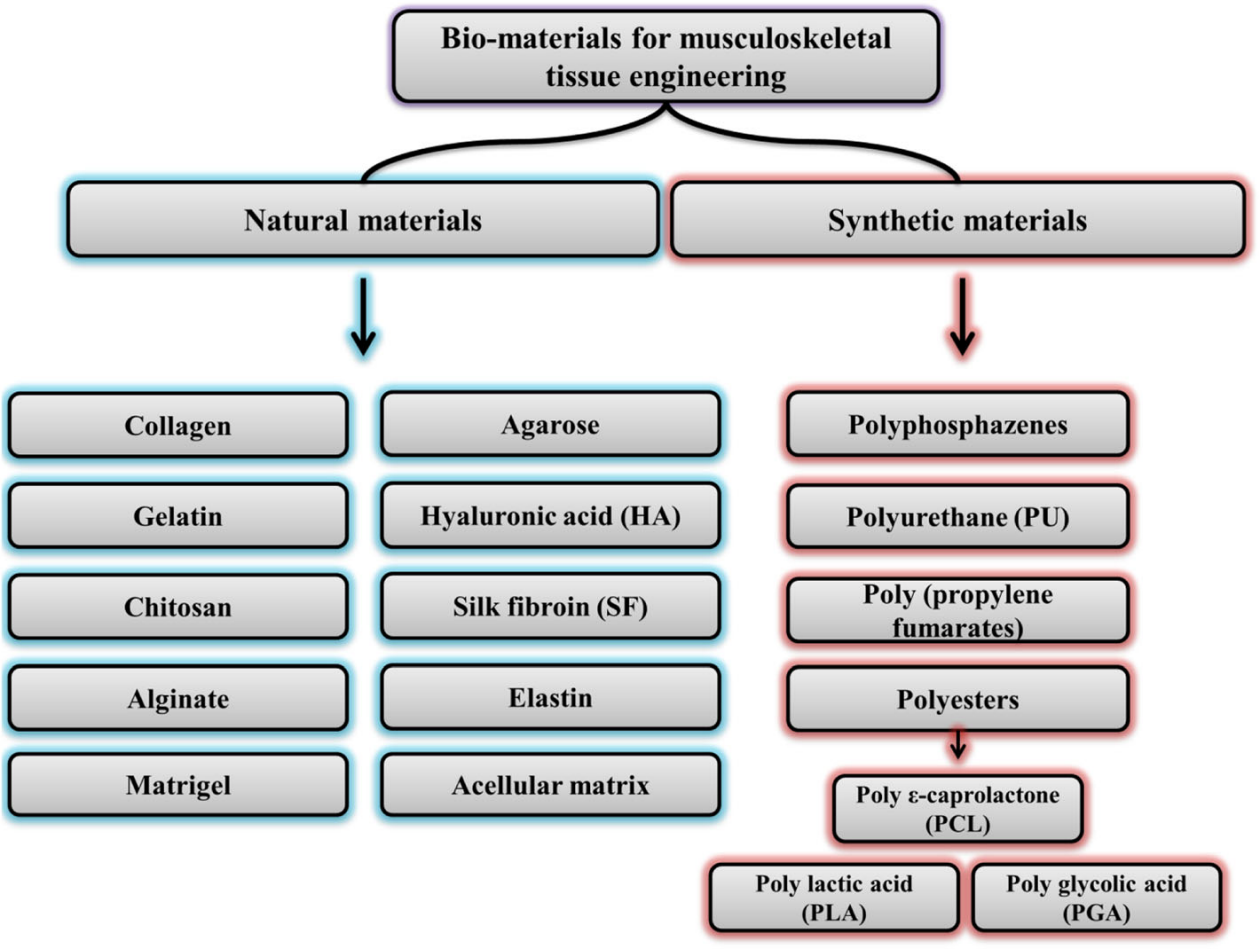
Common biomedical materials used in musculoskeletal tissue engineering, including natural and synthetic materials

The first line of treatment for musculoskeletal defects is autograft (taken from the patient) and allograft (taken from cadavers). Although this therapeutic approach has excellent advantages, including mechanical/ structural/ combination properties similar to host tissue, it has some disadvantages such as limited access to autografts and the transmission of diseases in allograft cases [26]. Therefore, the use of another therapeutic approach in the musculoskeletal system is suggested. In this regard, many advances have been made in the field of biomaterials and biomaterial-based methods to create engineered grafts for use in repairing damaged musculoskeletal tissues and reconstruct them. Since the tissues of the musculoskeletal system have a range of mechanical characteristics, to imitate these properties, various biomaterials with different mechanical and physical attributes have expanded. Common biomaterials which are used in musculoskeletal tissue engineering were presented in Fig. 1.

One of the significant challenges in the musculoskeletal system therapeutics is the repair of cartilage tissue problems because the ability to regenerate damaged cartilage tissue is limited [27]. One of the main ways to solve this problem is to use biomaterials [28]. Like other tissues in the musculoskeletal system, cartilage tissue also requires the use of biomaterials with specific characteristics. Biocompatibility, biodegradability, support for cellular proliferation and differentiation, the ability to transfer gases and nutrients and waste materials, and having appropriate mechanical properties are among the characteristics required for biomaterials to be used in cartilage tissue engineering [29]. Clinically, researchers in cartilage tissue engineering have used various biomaterials to repair or replace damaged cartilage tissue, which includes a variety of natural materials such as GAGs, polysaccharides, and different proteins and synthetic materials such as polyesters of poly(lactic-co-glycolic acid) (PLGA) family [30–32].

It should be noted that any biocompatible material used as a scaffold in musculoskeletal tissue engineering has a vital role in the behavior of stem cells, in particular, their proliferation and differentiation [33, 34]. During the tissue engineering process of the musculoskeletal system performed on scaffolds made of biocompatible and biomimetic materials, tissue-specific molecular markers are expressed, as shown in Table 1.

**Table 1:** Molecular markers of musculoskeletal tissues involved during the tissue engineering process on biocompatible and biomimetic materials

	Specific Markers	References
Chondrogenic markers	SRY-box 9 (SOX 9), Collagen type 1, Collagen type II, Collagen type III, Collagen type IXα3, Aggrecan, and a cartilage-specific proteoglycan	[35–38]
Myogenic markers	Desmin, myosin heavy chain 2, myocyte enhancer factor 2, Alpha actinin skeletal muscle 2 (ACTN2), MyoD, Myogenin (MYOG) and Troponin T	[39–41]
Osteogenic markers	Runt-related transcription factor 2 (Runx2), Collagen type I, Alkaline phosphatase (ALP), Osteocalcin (Ocn) and Osteopontin (Opn)	[42, 43]
Tenogenic markers	Collagen type I, Collagen type III, Scleraxis (SCX), Mohawk homeobox (Mkx), Tenomodulin (TNMD), Tenascin C, Biglycan, and Fibronectin	[42, 44, 45]
Ligamentogenic markers	Collagen type I, Collagen type III, Decorin, Biglycan and Aggrecan	[46, 47]

### Physical property of biomimetic biomaterials and musculoskeletal tissue engineering

To better imitate a defective tissue in musculoskeletal tissue engineering, materials with chemical and physical characteristics similar to the target tissue should be used. The three common types of biomaterials based on the biophysical properties used for the musculoskeletal system include flexible/ elastic, hard, and soft biomaterials as described below.

#### Flexible/ elastic biomaterials

In terms of mechanical properties, meniscus (M), tendon (T) and ligament (L) tissues are flexible in the musculoskeletal system and are considered as elastic tissues. M/T/L has a poor vascular system, so the oxygen and nutrients needed to repair and regenerate them are lower than other tissues [48]. Due to the low repair capacity in these tissues, in the event of injury, surgical procedures, including autografts and allografts, are required [49]. But because of the limitations of these methods, such as graft failure and morbidity, the engineering of M/T/L biomaterials is a promising method. Common biomimetic biomaterials for use in engineering of elastic tissues include collagen, elastin, PLLA, PU, and PCL [50, 51]. For example, a composite of Fiber/collagen has been used to create a structure with a high elastic property for use in ligament by Patrick et al. [52].

#### Hard biomaterials

Bone tissue is one of the significant components of the musculoskeletal system that requires hard materials to be resuscitated or engineered. In different orthopedic procedures, which increase each day, have been used various materials with their distinct advantages and disadvantages. The first hard biomaterials to use in hard tissues were ceramics and bio-glasses [53, 54]. Then, absorbable and biocompatible biomaterials such as calcium sulfate- and calcium phosphate-based materials appeared. Different combinations of calcium and phosphate for orthopedic applications, for example, as bone cement, have been studied [55, 56]. In addition, as a result of the degradation of these materials, sulfate, phosphate, and calcium are formed, which are part of the ions present in the body and are harmless in this regard. Of the different types of known calcium phosphate, hydroxyapatite (Ca_10_(PO_4_)_6_(OH)_2_) has been more prominent. Hence scientists have used various hydroxyapatite combinations with natural or synthetic biodegradable polymers for creating composite scaffolds that are usable in hard tissues (osteochondral and bone) [10, 57–59].

#### Soft biomaterials

Soft materials that contain some natural and synthetic biomaterials are used to construct structures for use in soft tissues of the musculoskeletal system such as muscle and cartilage. Common natural materials used for soft tissues of the musculoskeletal system include collagen, gelatin, hyaluronic acid, chitosan, and matrix acellular [60, 61]. Specifically, hydrogel structures and sponges made of alginate, agarose, collagen, hyaluronan, fibrin gels, poly (glycolic acid) (PGA) and poly (lactic acid) (PLA), are employed in cartilage tissue engineering [62].

### Natural polymers for musculoskeletal and cartilage tissue engineering

Natural polymers are employed extensively in tissue engineering due to biocompatibility, enzymatic degradation, and the ability to conjugate with various factors, such as growth factors [63, 64]. Of course, it is an advantage if the degree of enzymatic degradation of the polymer is controlled; otherwise, it is a disadvantage of natural polymers [65]. Also, batch-to-batch variability in purity and molecular weight is a disadvantage of biological polymers [66].

**Fig. 2:**
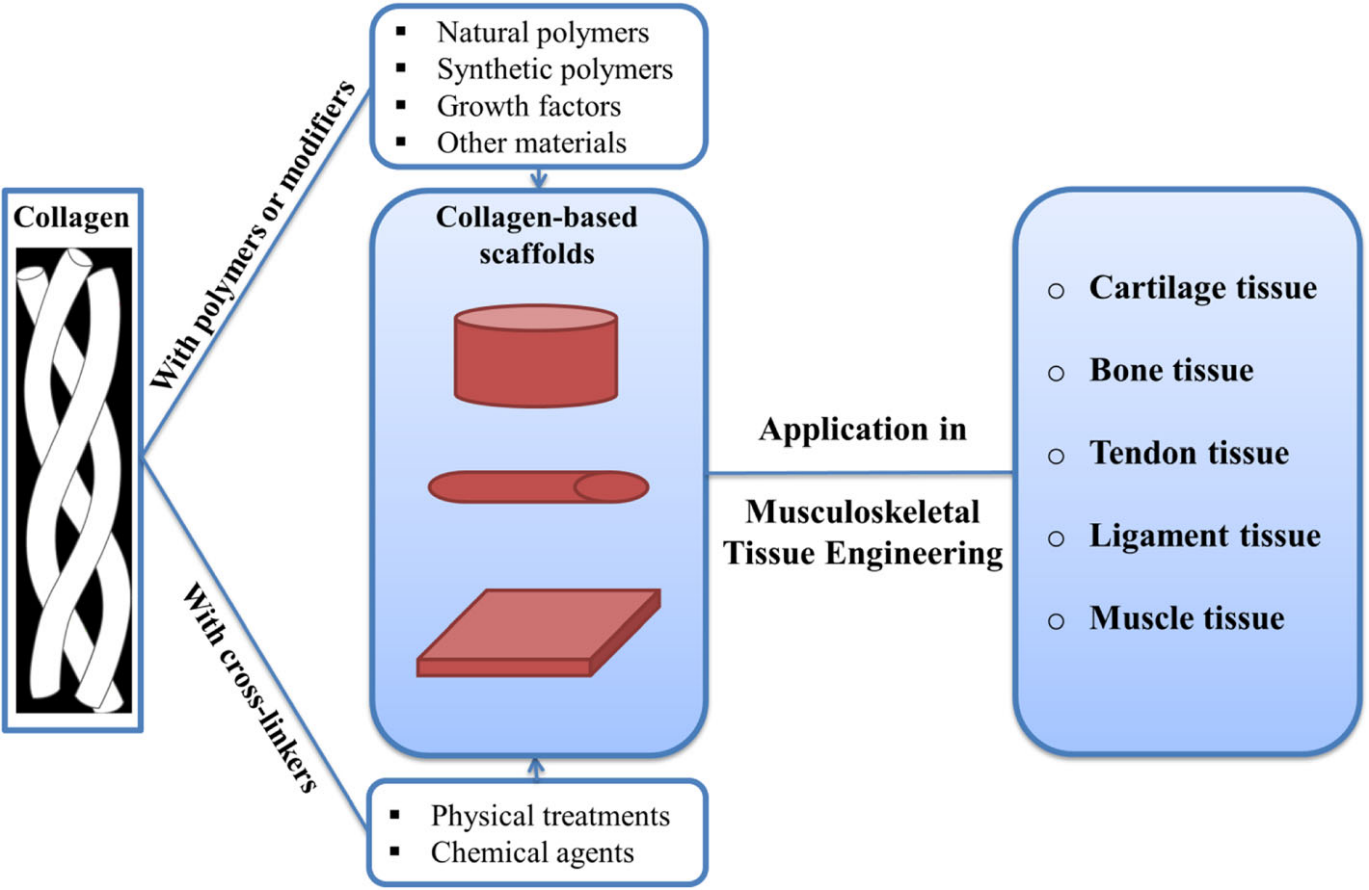
Collagen-based scaffolds in musculoskeletal tissue engineering

A wide range of natural polymers (biological polymers), including collagen, gelatin, chitosan, alginate, agarose, hyaluronic acid (HA), silk fibroin, elastin, matrigel, acellular matrix, and some other biological materials are used in the engineering of musculoskeletal tissues, including bone, tendon, meniscus, and muscle and cartilage. It has been proven that **collagen**, due to its many RGD residues (arginine, glycine and aspartate), can increase cell attachment and also help differentiate precursor cells into bone-forming cells [67]. Since collagen-based scaffolds have excellent properties such as biocompatibility, biodegradability, low immunogenicity, porous structure, and good permeability, have been widely used in musculoskeletal tissue engineering (Fig. 2).

Shangwu Chen et al. prepared 3D micro-grooved scaffolds based on collagen with big concave micro-grooves (about 120–380 μm) for skeletal muscle tissue engineering [12]. These researchers obtained highly aligned and multi-layered scaffold. It was observed that Myoblasts in the engineered muscle tissue were well-aligned with upper expression of myosin heavy chain and high construction of muscle ECM [12]. Because collagen can support cellular activities of mesenchymal stem cells (MSCs) and articular chondrocytes (ACs), and can be prepared as a hydrogel or solid scaffold, it is used extensively in cartilage tissue engineering [68]. Of the sixteen known types of collagen, types I, II, and III form the most considerable amount of collagen in the body, of which type II is the predominant type of collagen in cartilage tissue [69]. It should be noted that the behavior of chondrocytes is affected by the type of collagen present in the extracellular matrix [70]. For example, chondrocytes in the collagen type II retain their spherical phenotype better than when they are in the collagen type I [71]. On the other hand, although collagen type II mimics the natural environment of cartilage tissue better, collagen type I is often used in tissue engineering because it is easily separated by acetic acid solution as an animal by-product [72]. Also, collagen type I is capable of in situ polymerization at physiological temperature and neutral pH [32, 73]. Xingchen Yang et al. used sodium alginate (SA) with collagen type I (COL) as bio-inks for bio-printing and then incorporated chondrocytes to construct in vitro printed cartilage tissue [74]. Finally, the results showed that 3D printed structures have significantly improved mechanical strength compared to sodium alginate alone. It was also observed that SA/COL scaffold helped cell adhesion and proliferation and also increased the expression of cartilage-specific genes, including Sox9, Col2al, and Acan.

**Fig. 3:**
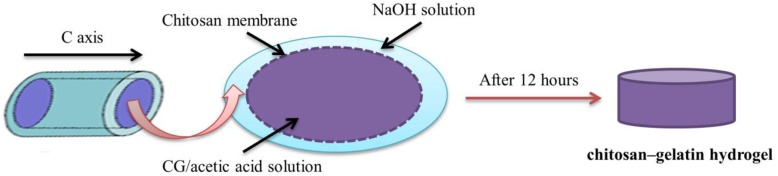
Schematic illustration of preparing of chitosan-gelatin gel through in situ precipitation method [77]

**Gelatin** as a biocompatible and biodegradable protein-based polymer is produced from collagen degradation. Gelatin due to having bioactive motifs (L-arginine, glycine, and L-aspartic acid (RGD) peptides) is a useful polymer for enhancing cell surface adhesion. The soluble nature of gelatin in the aqueous surroundings at human-body temperature (about 37 °C) is one of the limitations of using it in tissue engineering, so it is essential to cross-link with agents such as glutaraldehyde, water-soluble carbodiimide, and bis-epoxy. Covalent cross-linking in chemically cross-linked fiber can improve gelatin mechanical properties and stability [75]. Hydrogel scaffolds, based on gelatin and collagen due to their properties have attracted much attention in regenerative medicine [64]. Cells within gelatin/collagen hydrogels are homogeneously distributed during gel preparation [9]. This demonstrates the best ability of this hydrogels to create tissue for use in tissue engineering. There are various methods for cross-linking of gelatin and collagen. Chemical approaches, such as using aldehydes are often toxic. Another cross-linker is genipin that improves the mechanical characteristics of gelatin and collagen [8]. Also, electrospinning is the most suitable method for preparing Nano-fibrous networks, which can mimic the native ECM of tissues [10]. The electrospun Nano-fiber scaffolds have advantages such as high surface to volume ratio and high porosity that is appropriate for cell attachment, cell communication, as well as for nutrient transporting [10]. Various nanofibers have been used for cartilage tissue engineering, but most of them because of the small pore size and low thickness, did not support 3D cartilage regeneration. On the other hand, the fabrication of 3D Nano-fibrous scaffolds is a challenge. Weiming Chen et al. fabricated an electrospun gelatin/PLA nanofiber as a porous 3D scaffold for cartilage tissue engineering [76]. They also modified the structures with hyaluronic acid to improve the repair effect in the cartilage. Results showed that scaffolds were superabsorbent and cytocompatible [76]. In another work done by Zhi-Sen Shen et al. for cartilage tissue engineering, the chitosan-gelatin (CG) gel was made with in situ precipitation process [77], as shown in Fig. 3. In this method, the chitosan membrane was first filled with a solution of CG / acetic acid and then placed in a NaOH solution. After 12 h, the gel forms through the penetration of OH from the NaOH to the c axis.

**Fig. 4:**
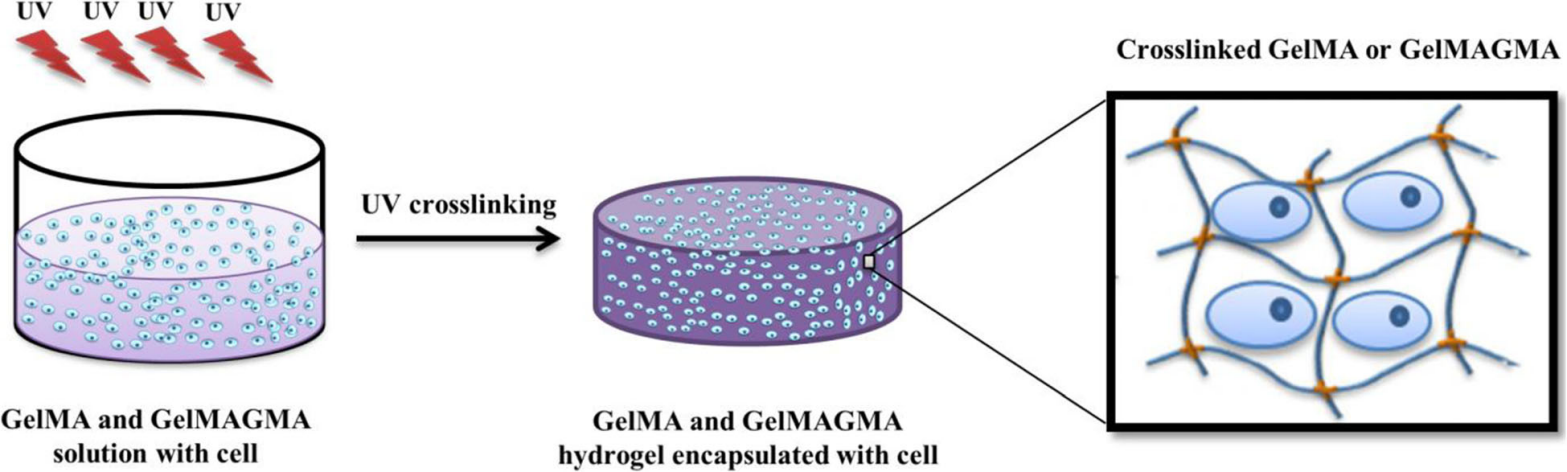
Schematic illustration of preparing of GelMA and GelMAGMA hydrogel loaded with the cell for cartilage tissue engineering [80]

Gelatin methacrylate (GelMA) hydrogel is another type of gel that has been used for reconstruction of various tissues, especially cartilage, due to its injectability and biocompatibility [78, 79]. Nevertheless, weak mechanical properties and rapid degeneration are the disadvantages of GelMA hydrogels that need to be improved [79]. For this purpose, Xiaomeng Li et al. made double modified gelatin so that they used methacrylic anhydride and glycidyl methacrylate to activate amino groups and hydroxyl/ carboxyl groups in gelatin, respectively [80]. The modified gelatin macromers in this work are known as GelMA and GelMAGMA, respectively. They then used double modified gelatin to prepare high crosslinking density hydrogels. In this way, Chondrocytes were placed in a macromer solution, and then UV irradiation was used to prepare a cell-laden hydrogel (Fig. 4).

Of course, it should be noted that gelatin due to its highly hydrophilic surface and the fast degradation time may not be suitable as a base material for scaffolds. To improve the properties of gelatin-based structures, blending it with other polymers such as PCL can be better. Ke Ren et al. fabricated a composite nanofiber scaffold based on PCL and gelatin using genipin for bone tissue. Results demonstrated the incorporation of gelatin into PCL nanofibers improved the cell adhesion, viability, proliferation, and osteogenic capability. Also, crosslinking by genipin enhanced the tensile properties of nanofibers that are important for bone regeneration [81].

**Fig. 5:**
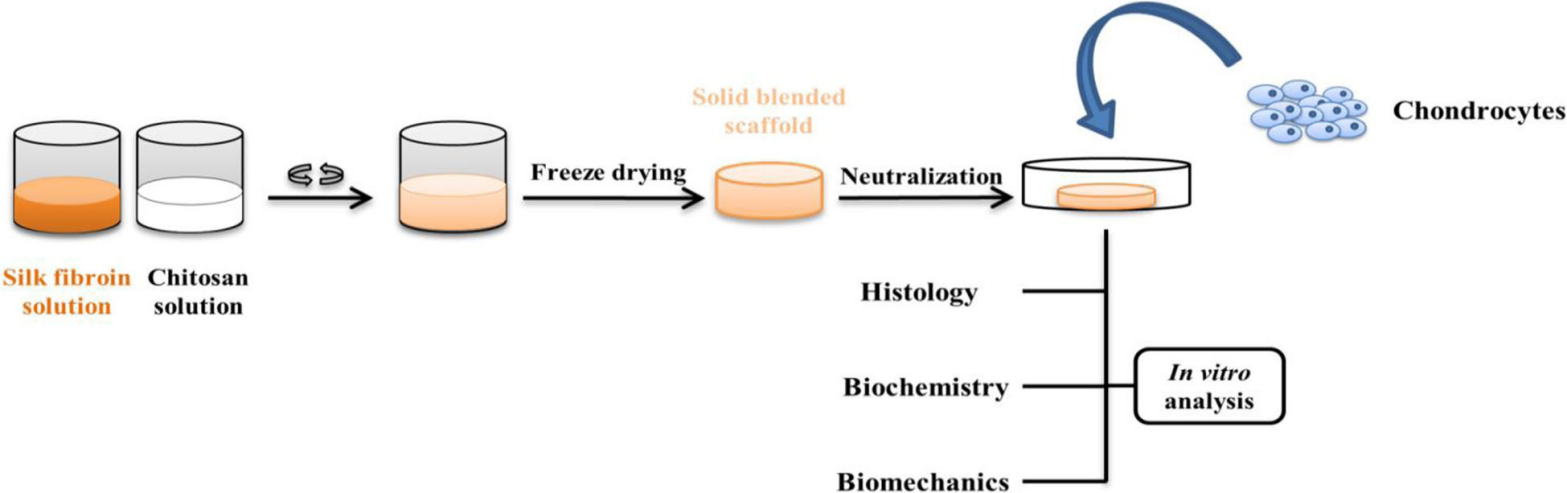
Schematic illustration of the experimental design of 3D silk fibroin/chitosan scaffolds for cartilage tissue engineering [84]

**Chitosan**, as an antimicrobial polymer, which is derived from chitin, is a linear polysaccharide. The components of chitosan are glucosamine and N-acetyl-glucosamine. This type of natural polymer due to its excellent properties such as biocompatibility and biodegradability has been considered as a useful biomaterial in tissue engineering [82]. Chitosan, because of many primary amines can form ionic complexes with anionic polymers or can be modified with different types of cross-linkable groups [67]. Also, chitosan due to its structural similarity to the main part of the native ECM of the cartilage and bone (glycosaminoglycan) has attracted considerable interest [83]. Chitosan hydrogels can be modified with different agents to create a favorable osteogenic environment. Christopher Arakawa et al. fabricated a composite scaffold based on photopolymerizable methacrylated glycol chitosan (MeGC) hydrogel containing collagen (Col) with a riboflavin photo-initiator to bone tissue engineering [67]. In this study, incorporation of Col in MeGC-based hydrogels slowed the degradation rate and increased the compressive modulus of these hydrogels. Also, the prepared composite hydrogels improved cellular behaviors, including attachment, proliferation, and osteogenic differentiation [67]. In a study, YiminHu et al. made a cross-linked composite scaffold containing chondroitin sulfate, hyaluronic acid, nano-hydroxyapatite (nHAP) and chitosan [83]. Chondroitin sulfate is a sulfated glycosaminoglycan and is one of the ECM components of cartilage and other tissues. Chondroitin sulfate because of its excellent properties such as biological activity, anti-inflammatory activity and inhibition the cartilage degradation, which is carried out by inhibiting the production of enzymes responsible for degradation, has been considered in cartilage repair. Also, both hyaluronic acid and chondroitin sulfate due to their negative charges retain water in the cartilage tissue. Finally, results indicated that composite scaffolds had appropriate mechanical strength because of the addition of the nHAP and interaction between the positive charge of chitosan and the negative charge of hyaluronic acid and chondroitin sulfate. It was also illustrated that these scaffolds improved the proliferation and differentiation of osteoblast [83]. As already mentioned, Chitosan is effective material in repairing cartilage due to its structural similarity to glycosaminoglycans. In this regard, to use chitosan-based natural scaffolds instead of synthetic scaffolds for the cartilage tissue engineering, Nandana Bhardwaj constructed 3D silk fibroin/chitosan scaffolds loaded with bovine chondrocytes (Fig. 5) [84]. The results showed that these scaffolds had unique viscoelastic properties that are very important for cartilage tissue.

**Fig. 6:**
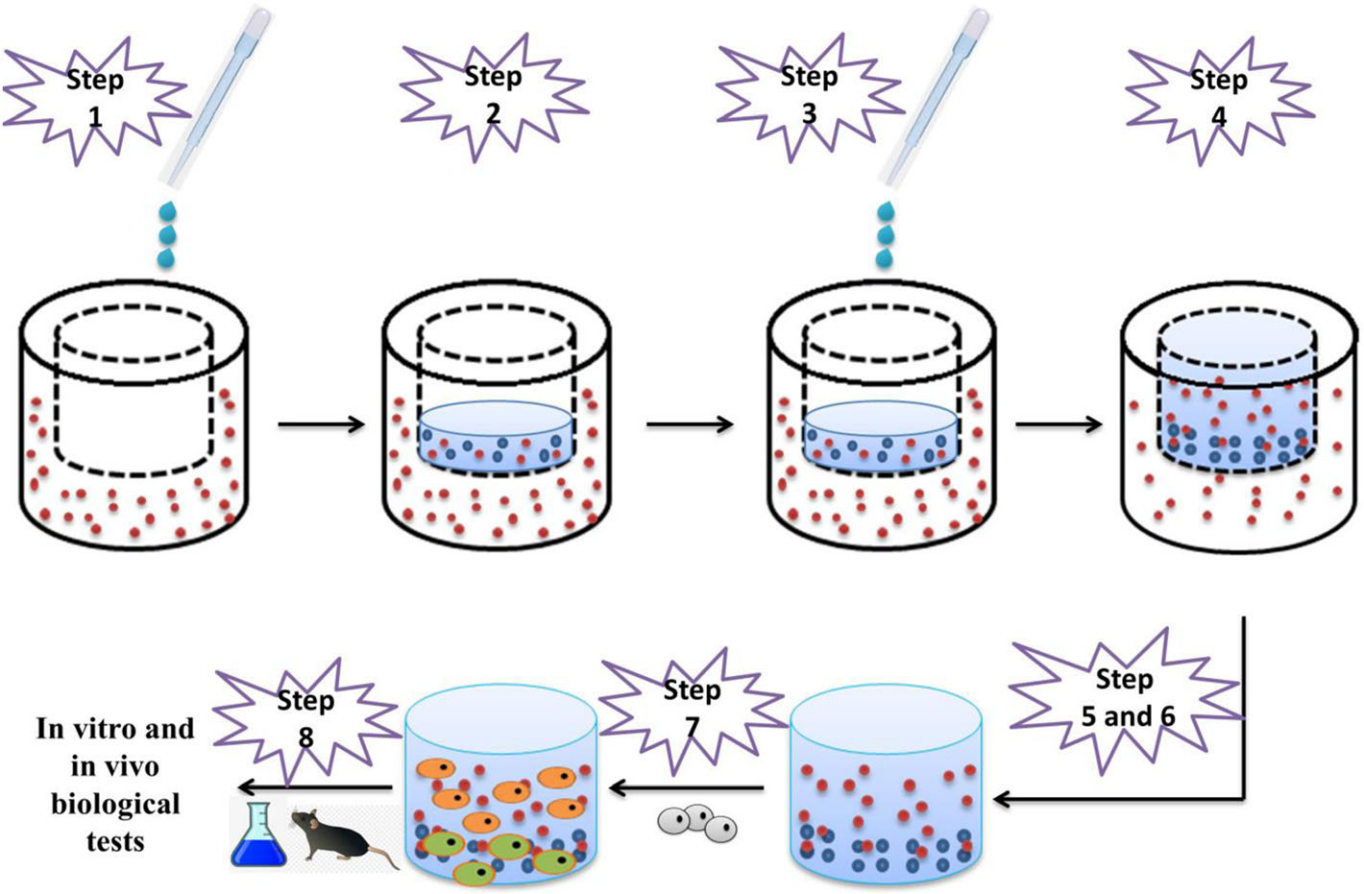
Schematic illustration of the process of preparing an alginate-based bilayered scaffold for cartilage tissue engineering [89]. **Step 1:** introduction of alginate solution + HA into the agar mold. **Step 2:** gelation of the bony layer by Ca^2+^ crosslinking. **Step 3:** introduction of alginate sulfate solution + TGF- β1. **Step 4:** gelation of the chondral layer by Ca^2+^ crosslinking. **Step 5 and 6**: removal of the monolithic hydrogel and freeze-drying. **Step 7:** cell seeding. **Step 8:** biological tests

**Alginate** is another natural polysaccharide that is extracted from brown sea algae, and consists of (1 → 4) linked β-Dmannuronate (M) and α-L-guluronate (G) residues [85]. Alginate is easily cross-linked through a rapid reaction between calcium cations and carboxyl groups of alginate [86]. But, the direct introduction of calcium cations in alginate solution because of its fast reaction cannot make a symmetrical hydrogel [87]. In the recent years, a novel technique has been advanced for the fabrication of homogeneous alginate hydrogel based on slowly releasing calcium cations from CaCO3 through its reaction with protons derived from hydrolysis of glucono-d-lactone (GDL) [7]. Alginate-based hydrogels are widely used in cartilage tissue engineering. In one of these studies, conducted by JinFeng Liao et al., injectable 3D alginate hydrogel was made that was loaded with poly(ε-caprolactone) − b-poly-(ethylene glycol) − b-poly(ε-caprolactone) microspheres (MPs/Alg) [88]. In the suspension of chondrocytes/alginate and porous microspheres, due to calcium gluconate release, a gel was formed that affect the repair of cartilage tissue. In another work done for osteochondral tissue repair, Luca Coluccino et al. constructed a bioactive scaffold based on alginate and transforming growth factor-β (TGF- β1)/hydroxyapatite (HA) (Fig. 6) [89]. They made porous alginate scaffolds through the freeze-drying of calcium cross-linked alginates. They also used TGF and HA as bioactive signals to offer a chondroinductive and osteoinductive surface. Finally, the results showed that the designed scaffold is promising for osteochondral tissue engineering.

**Fig. 7:**
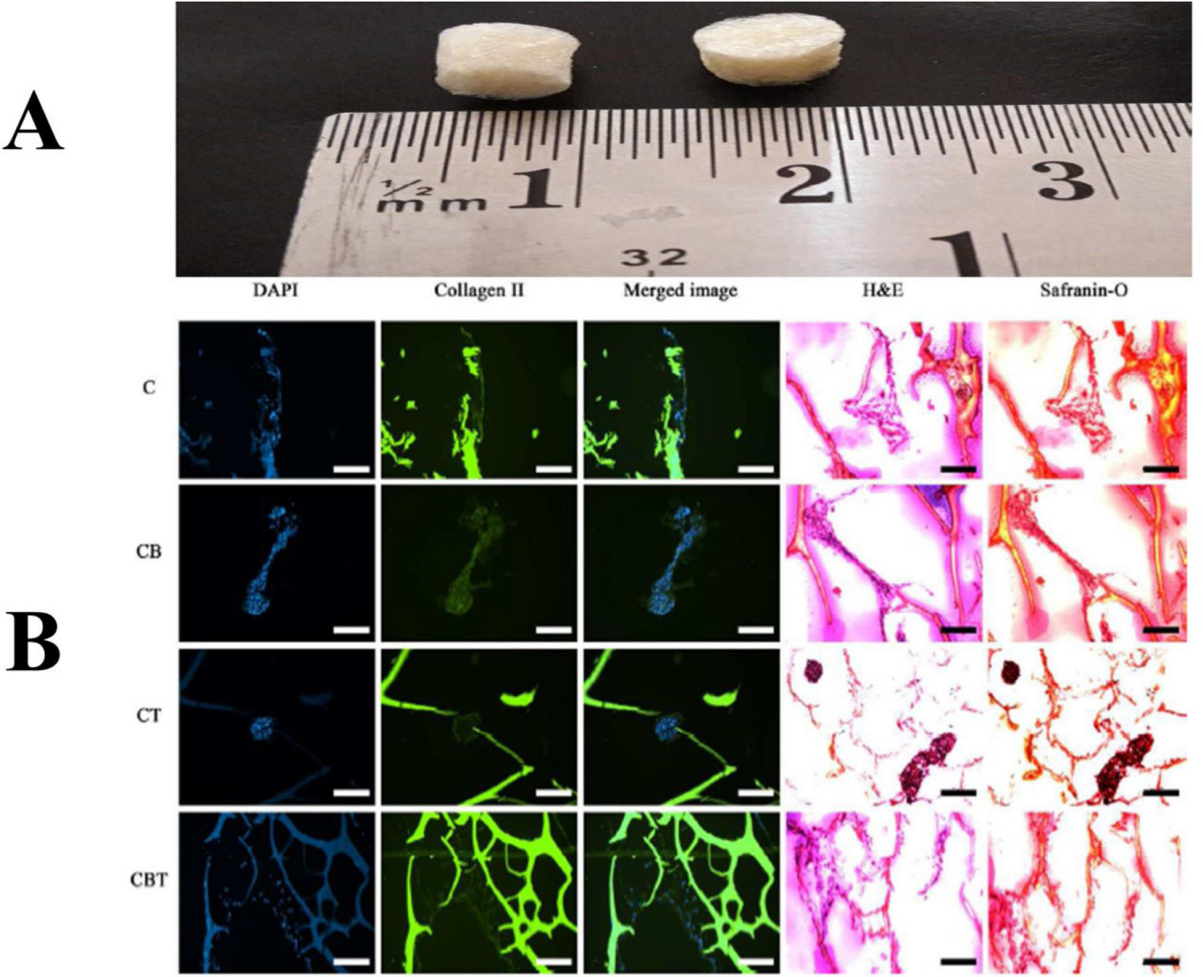
(**a**) Macroscopic image of chitosan-agarose (CHAG) scaffolds. (**b**) Histological examination of HWJ-MSCs on the CHAG scaffolds in chondrogenic medium, with or without growth factors TGFβ3 and BMP-2. Immunostaining was done with DAPI, collagen-II + FITC, merged image, and also hematoxylin and eosin (H&E) staining and Safranin-O staining for sGAG was done. Groups cod: C) chondrogenic medium alone, CB) chondrogenic medium with BMP-2, CT) chondrogenic medium with TGFβ3, CBT) chondrogenic medium with BMP-2 and TGFβ3. Scale bars represent 100 μm. Republished with permission of ref. [93], Merlin Rajesh Lal L, Suraishkumar G, Nair PD. Chitosan-agarose scaffolds supports chondrogenesis of Human Wharton’s Jelly mesenchymal stem cells. Journal of Biomedical Materials Research Part A. 2017;105(7):1845–55, Copyright (2019)

**Agarose** is a natural, transparent, and neutrally charged polysaccharide that widely used in cartilage tissue engineering [90, 91]. Also, this polymer has applied as a scaffold for autologous chondrocyte implantation strategy [90]. In previous studies, it has been demonstrated that agarose hydrogel can be mechanically suitable for long-term culturing of chondrocyte [92]. However, agarose has some drawbacks such as small cell adhesiveness, low cell proliferation, and little graft integration with the host tissue. So, it seems that the combination of agarose with other polymers such as gelatin and chitosan can be better [91]. For example, Merlin Rajesh Lal LP et al. fabricated a chitosan-agarose (CHAG) scaffold that mimics the native cartilage extracellular matrix [93]. They then cultured the Human Wharton’s Jelly Mesenchymal Stem Cells (HWJMSCs) on the CHAG scaffolds in a chondrogenic medium. Their results indicated that these scaffolds are useful in repairing the cartilage tissue (Fig. 7).

**Fig. 8:**
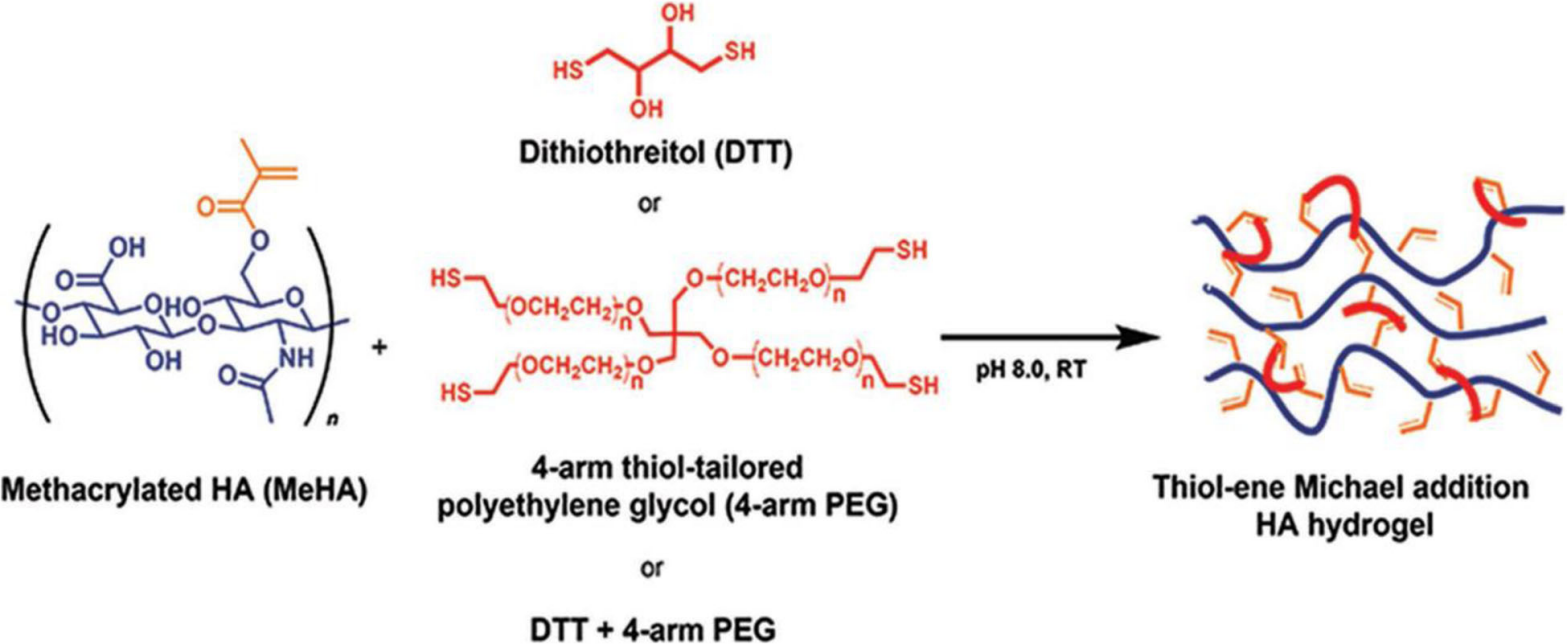
Formation of HA-based hydrogels through the reaction between thiol-based crosslinkers and methacrylate groups on HA. Republished with permission of ref. [98], Yuan J, Maturavongsadit P, Metavarayuth K, Luckanagul JA, Wang Q. Enhanced Bone Defect Repair by Polymeric Substitute Fillers of MultiArm Polyethylene Glycol-Crosslinked Hyaluronic Acid Hydrogels. Macromolecular bioscience. 2019:1900021, Copyright (2019)

**Hyaluronan (HA)** is known as an anionic polysaccharide that has been studied abundantly to improve cartilage repair. HA because of poor mechanical properties, even after cross-linking, cannot be used alone to make scaffolds. To print 3D structures, HA usually functionalized with UV-curable methacrylate [94]. However, using photo-initiators and acrylate-based monomers can be toxic [95]. Kun-CheHung et al. fabricated 3D printed structures based on water-based polyurethane (PU) elastic nanoparticles, bioactive components, and hyaluronan [96]. The water-based system can enhance the bioactivity of the growth factor/ drug encapsulated in the printed scaffolds. The results showed that these printed scaffolds could timely release the bioactive molecules, improve the self-aggregation of mesenchymal stem cells, stimulate the chondrogenic differentiation of MSCs, and increase the production of ECM for cartilage repair [96]. Hyaluronic acid, as an injectable hydrogel, is widely used for various tissues of the musculoskeletal system, especially the cartilage tissue [97–99]. In many studies for cartilage tissue, hyaluronic acid-based hydrogels have been used as a cell delivery system for cartilage regeneration [97, 100, 101]. For example, in a study conducted by Elaheh Jooybar et al. for cartilage regeneration, the human mesenchymal stem cell (hMSCs)-laden in the injectable hyaluronic acid-tyramine (HA-TA) hydrogel was used, and the platelet lysate (PL) was incorporated into it as an inexpensive and autologous source of growth factors [97]. Finally, the results showed that the HA-TA-PL hydrogel induced the formation and deposition of cartilage-like extracellular matrix. Also, to enhance the osteogenesis of MSCs, Jishan Yuan et al. used hydrogels based on the multiarm polyethylene glycol (PEG) cross-linked with hyaluronic acid (HA) (PEG-HA hydrogels) [98]. Synthesis of three types of the HA-based hydrogels through Michael addition reaction between a thiol group of crosslinkers and methacrylate groups on HA is shown in Fig. 8. The results of a study by Jishan Yuan et al. showed that PEG-HA hydrogels are promising in bone regeneration.

Also, to improve the treatment of Volumetric muscle loss (VML), Juan Martin Silva Garcia et al. used the hyaluronic acid to make hydrogels that imitate the biomechanical and biochemical properties of the extracellular matrix of myogenic precursor and connective tissue cells [99]. For this purpose, they used poly(ethylene glycol) diacrylate and thiol-modified HA, and also used peptides such as laminin, fibronectin, and tenascin-C to functionalize them. The results showed that functionalized HA hydrogel with laminin peptide showed a better improvement in myogenic cell behaviors compared to other groups.

**Fig. 9:**
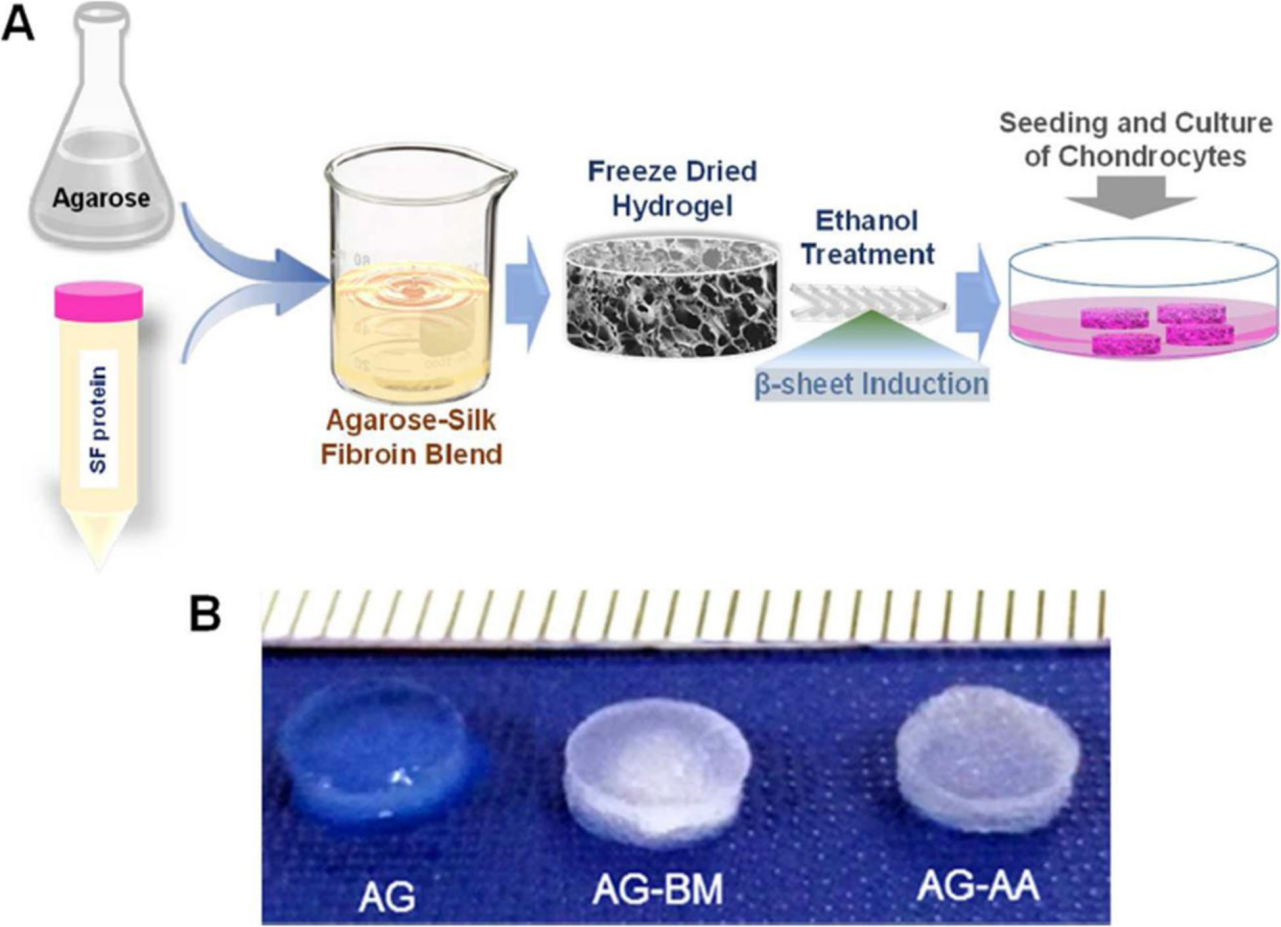
(**a**) Schematic illustration of the fabrication of silk fibroin hydrogel and (**b**) macroscopic image for cartilage tissue engineering. Republished with permission of ref. [91], Singh YP, Bhardwaj N, Mandal BB. Potential of Agarose/Silk Fibroin Blended Hydrogel for in Vitro Cartilage Tissue Engineering. ACS Applied Materials & Interfaces. 2016;8(33):21236–49, Copyright (2019)

**Silk fibroin** as a natural fibrous protein has some properties, for example, biocompatibility, biodegradability, tunable mechanical characteristics and fabrication into different formats (hydrogel, film, fiber, electrospun mats, porous scaffold, etc.) that make it usable for tissue engineering. Also, the similarity of silk hydrogel to ECM, lead to promising results in the field of tissue engineering. SF is employed as a scaffold for cartilage, bone, and ligament tissue engineering [91].. Nadine Matthias et al. worked on the volumetric muscle defect [102]. This type of muscle defect causes severe fibrosis if not treated. The purpose of the researchers in this work was to use stem cells combined with a biocompatible scaffold to repair muscle. To this end, they used muscle-derived stem cells (MDSCs) and a novel fibrin-based in situ gel casting. Finally, Nadine Matthias et al. showed that MDSCs can form new myofibers if cast with fibrin gel. It has also been shown that labeled cells with a LacZ can differentiate into new myofibers and increase muscle mass efficiently. Also, scaffold deposition and recovery of muscle ECM were determined by laminin and LacZ staining. Ultimately, complete repair of the damaged muscle was observed with MDSC/fibrin gel combination confirmed by immune-staining of striated myofiber marker (MYH1). In another work done by Sònia Font Tellado et al. to imitate the collagen alignment of the interface, the biphasic silk fibroin scaffolds with two different pore alignments, including anisotropic and isotropic, were made for tendon/ ligament and bone sides, respectively [103]. They finally demonstrated these biphasic silk fibroin scaffolds because of their unique properties, including stimulating effects on the gene expression of human adipose-derived mesenchymal stem cells (Ad MSCs) and better mechanical behavior, can be used in tendon/ligament-to-bone tissue engineering. Silk fibroin has been used extensively in the cartilage tissue engineering. For example, Yogendra Pratap Singh et al. fabricated the blend of silk fibroin and agarose hydrogels for cartilage tissue (Fig. 9) [91]. Auricular chondrocytes encapsulated in the blend hydrogel exhibited higher GAGs and collagen production. The results suggested that the blended hydrogels improved ECM production and cellular proliferation.

**Elastin** is the second part of the ECM that is responsible for helping the elasticity of many living tissues [104]. Elastin is an abundant protein in some tissues of the musculoskeletal system, including ligaments, tendon, and elastic cartilage. Hence, elastin has been studied abundantly in musculoskeletal tissue engineering [105]. Since 50% of elastic ligaments and 4% of tendons are from elastin, this protein is used in the studies related to the ligament and tendon tissues [106]. Helena Almeida et al. used tropoelastin to increase the stem cell tenogenic commitment in the tendon biomimetic scaffolds [105]. For this purpose, they constructed tendon biomimetic scaffolds using poly-ε-caprolactone, chitosan, and cellulose nanocrystals and then coated them with tropoelastin (TROPO) through polydopamine linking (PDA). The results showed that the combination of these scaffolds could modulate the stem cell tenogenic commitment and elastin-rich ECM production. Elastin-based scaffolds have also been used in cartilage engineering [107]. Annabi et al. prepared composite scaffold made of elastin and poly-caprolactone, which eventually porous scaffolds with improved biological and mechanical properties were obtained [108]. In vitro studies indicated that (PCL)/elastin scaffolds can support chondrocyte behaviors, including their adhesion and proliferation. Therefore, these composites have a high ability to repair the cartilage.

**Fig. 10:**
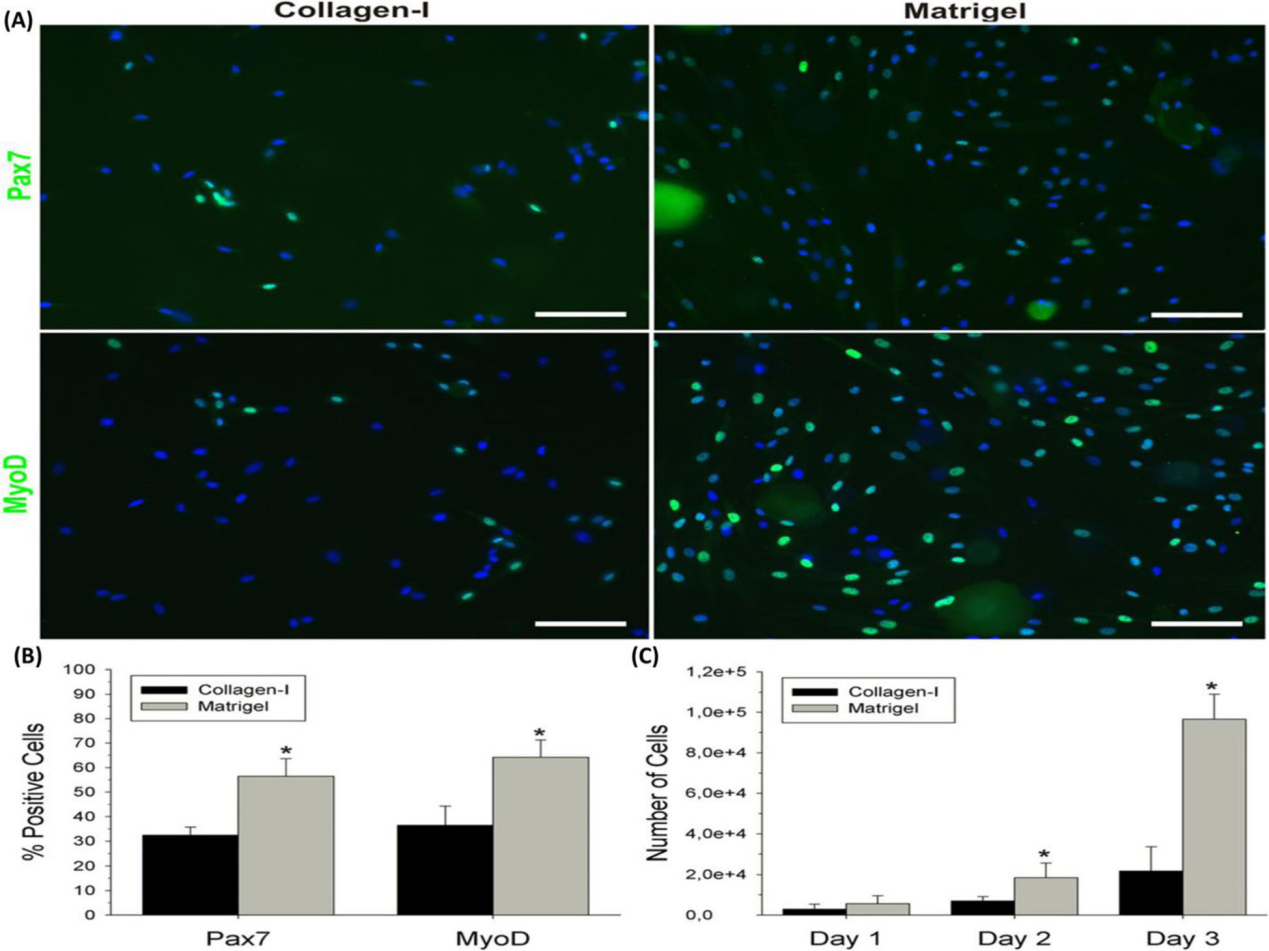
Fluorescent immunocytochemistry tests and quantification of Pax7 and MyoD. (**a**) Muscle stem cells in Matrigel and collagen-I coatings were stained for Pax7 or MyoD (both green) and DAPI (blue). (**b**) Quantification of Pax7^+^ and MyoD^+^ cells (expressed as a mean ± SD) in Matrigel and collagen-I coatings. (**c**) Indirect quantification of the number of cells (expressed as a mean ± SD) in Matrigel and collagen-I coatings. Scale bar represents 100 μm. ^∗^ Significant difference between collagen-I and Matrigel. Republished with permission of ref. [112], Grefte S, Vullinghs S, Kuijpers-Jagtman A, Torensma R, Von den Hoff J. Matrigel, but not collagen I, maintains the differentiation capacity of muscle-derived cells in vitro. Biomedical materials. 2012;7(5):055004, Copyright (2019)

**Fig. 11:**
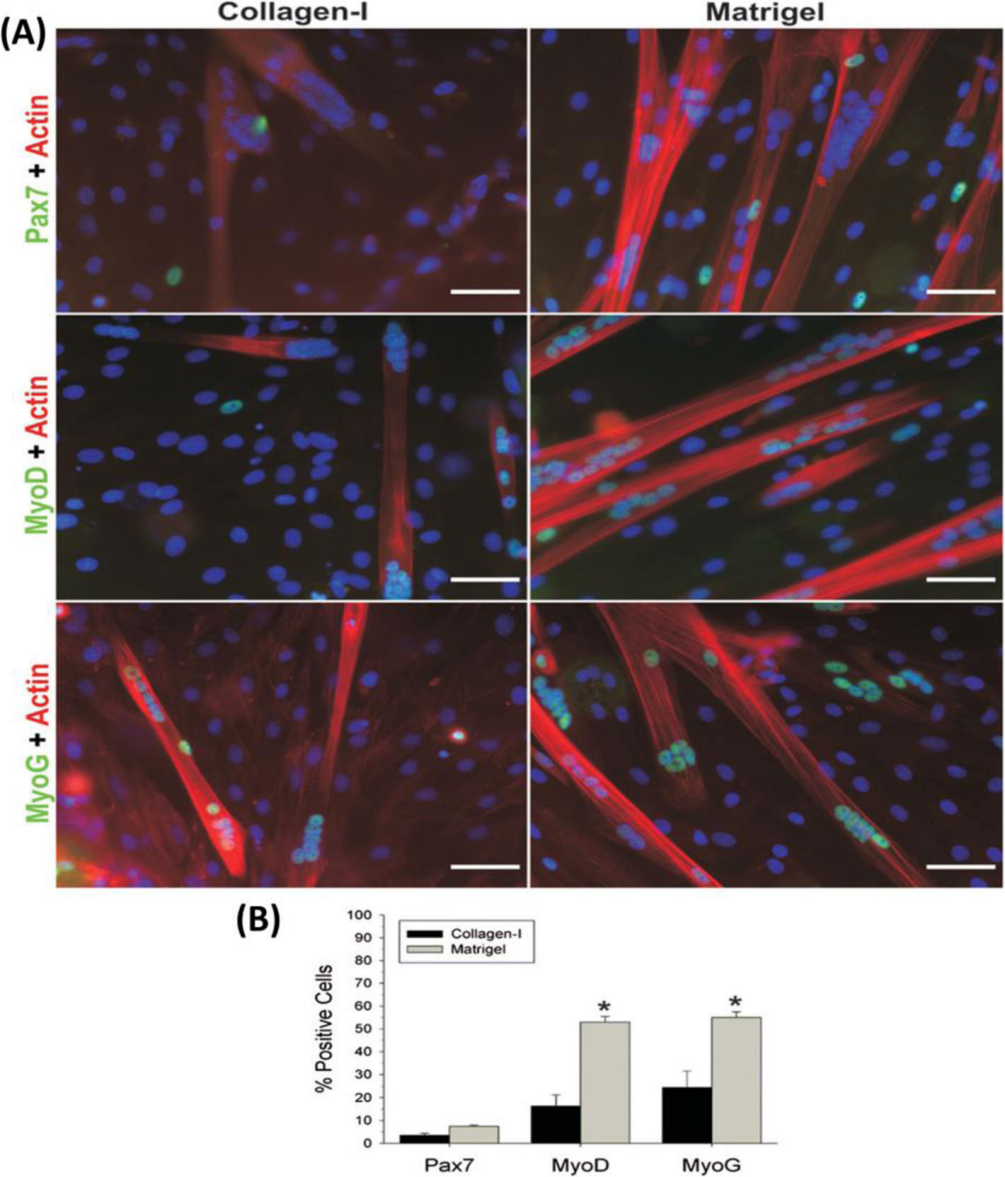
Fluorescent immunocytochemistry tests and quantification of Pax7, MyoD, and myogenin. (**a**) Muscle stem cells in Matrigel and collagen-I coatings were stained for Pax7, MyoD, or myogenin (all green) together with actin (red) and DAPI (blue) after differentiation. (**b**) Quantification of Pax7^+^, MyoD^+^, and myogenin^+^ cells (expressed as a mean ± SD) in Matrigel and collagen-I coatings after differentiation. Scale bar represents 50 μm. ^∗^ Significant difference between the Matrigel and collagen-I. Republished with permission of ref. [112], Grefte S, Vullinghs S, Kuijpers-Jagtman A, Torensma R, Von den Hoff J. Matrigel, but not collagen I, maintains the differentiation capacity of muscle-derived cells in vitro. Biomedical materials. 2012;7(5):055004, Copyright (2019)

**Matrigel** is another biological material used in the studies of the musculoskeletal system. The Matrigel matrix is extracted from mouse tumors and is a soluble form of basement membrane [109]. Matrigel contains various components of ECM proteins including laminin, collagen IV, entactin, and heparan sulfate proteoglycans. Therefore, Matrigel is used as a 3D model for studying cellular behavior [110, 111]. Grefte et al. studied differentiation and proliferation capacity of muscle stem cells in Matrigel or collagen type I gels. They proved the cellular behaviors of muscle precursor cells (proliferation and differentiation) in the Matrigel environment is more than the collagen environment (Figs. 10 and 11) [112].

In the past few years, Matrigel has also shown excellent performance in animal experiments for cartilage repair [113, 114]. Xiaopeng Xia et al. used Matrigel and chitosan/glycerophosphate (C/GP) gel to repair cartilage defects [113]. To do this, they incorporated transfected-chondrocyte cells with adenovirus holding BMP7 and green fluorescent protein (Ad-hBMP7-GFP) in both types of gel. They then transplanted the gels containing the chondrocytes into the rabbits’ knees, and after four weeks they examined the results. The results showed that the Matrigel containing Ad.hBMP7.GFP transfected chondrocytes successfully increased the repair of cartilage defects in the rabbit’s knee [113].

**Fig. 12:**
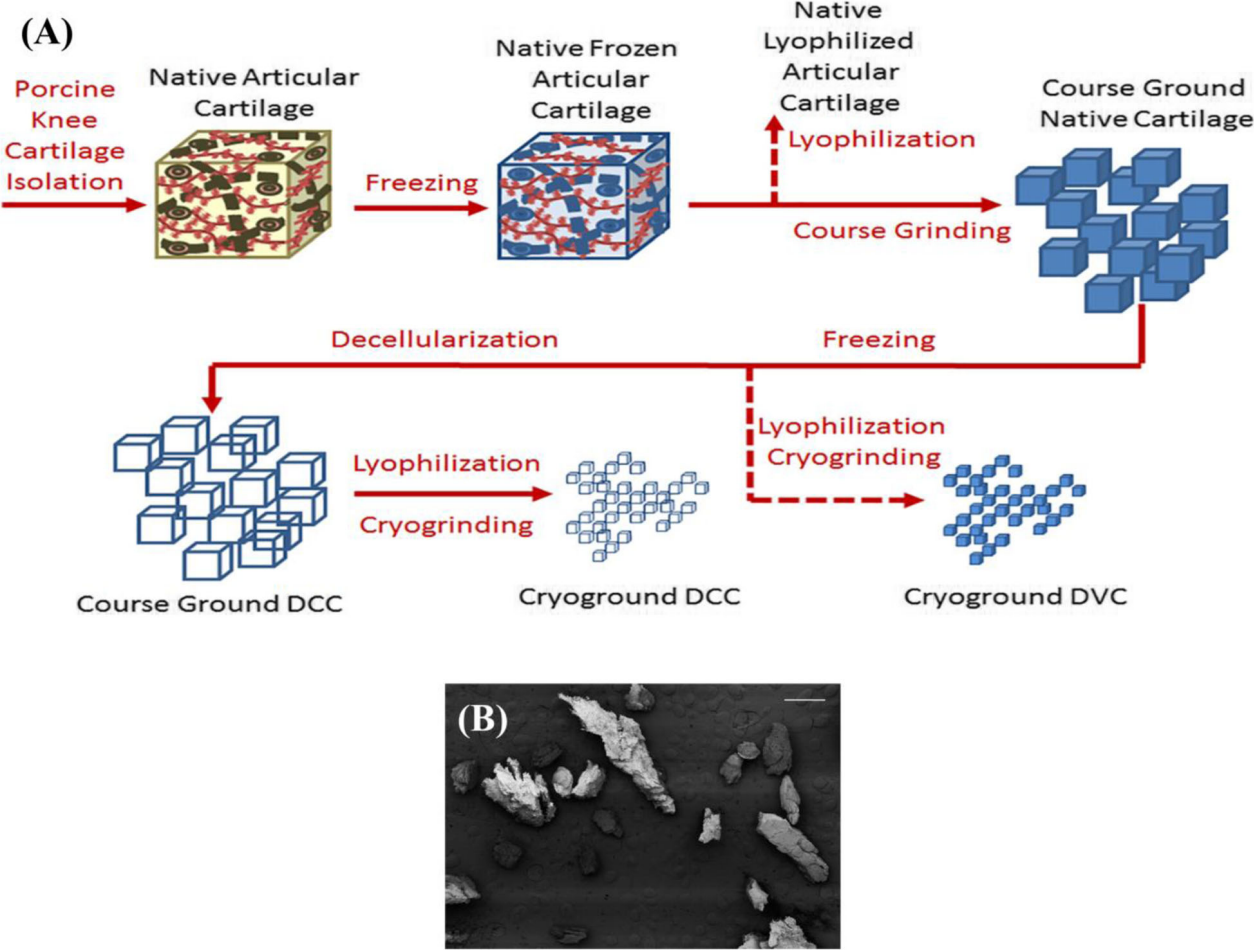
(**a**) Schematic illustration of Porcine Cartilage Processing. (**b**) SEM Image of Cryo-ground DCC. The scale bar is 1 mm. Republished with permission of ref. [130], Sutherland AJ, Beck EC, Dennis SC, Converse GL, Hopkins RA, Berkland CJ, et al. Decellularized cartilage may be a chondroinductive material for osteochondral tissue engineering. PloS one. 2015;10(5):e0121966, Copyright (2019)

An **acellular matrix** transplantation is a promising therapy for different tissues of musculoskeletal systems, especially for the treatment of injuries of muscles [115–117]. This type of biocompatible scaffold as a preformed and native ECM has also been used for bone, osteochondral, and articular cartilage defects [118–121]. Since the scaffolds based on the acellular matrix have mechanical properties and environment similar to the native tissue that is being repaired, the adhesion and migration of satellite cell are well done on them [122–127]. In a study, C2C12 cells were seeded on the intestine-derived biocompatible scaffold and then implanted in the rat for treating of volumetric muscle loss (VML) injury. After thirty-five days, the muscle fiber structure was observed by immunohistochemical staining [128]. In another study, small intestine submucosa (SIS)–ECM were used to repair muscle with bone fractures, which ultimately showed improvement in the repair process [129]. Amanda J. Sutherland et al. established a chemical decellularization process for articular cartilage tissue (Fig. 12) [130]. They constructed the chemically decellularized cartilage particles (DCC) and then cultivated rat bone marrow-derived mesenchymal stem cells (rBMSCs) on them. They then observed that the DCC had significantly increased chondroinduction of rBMSCs.

**Fig. 13:**
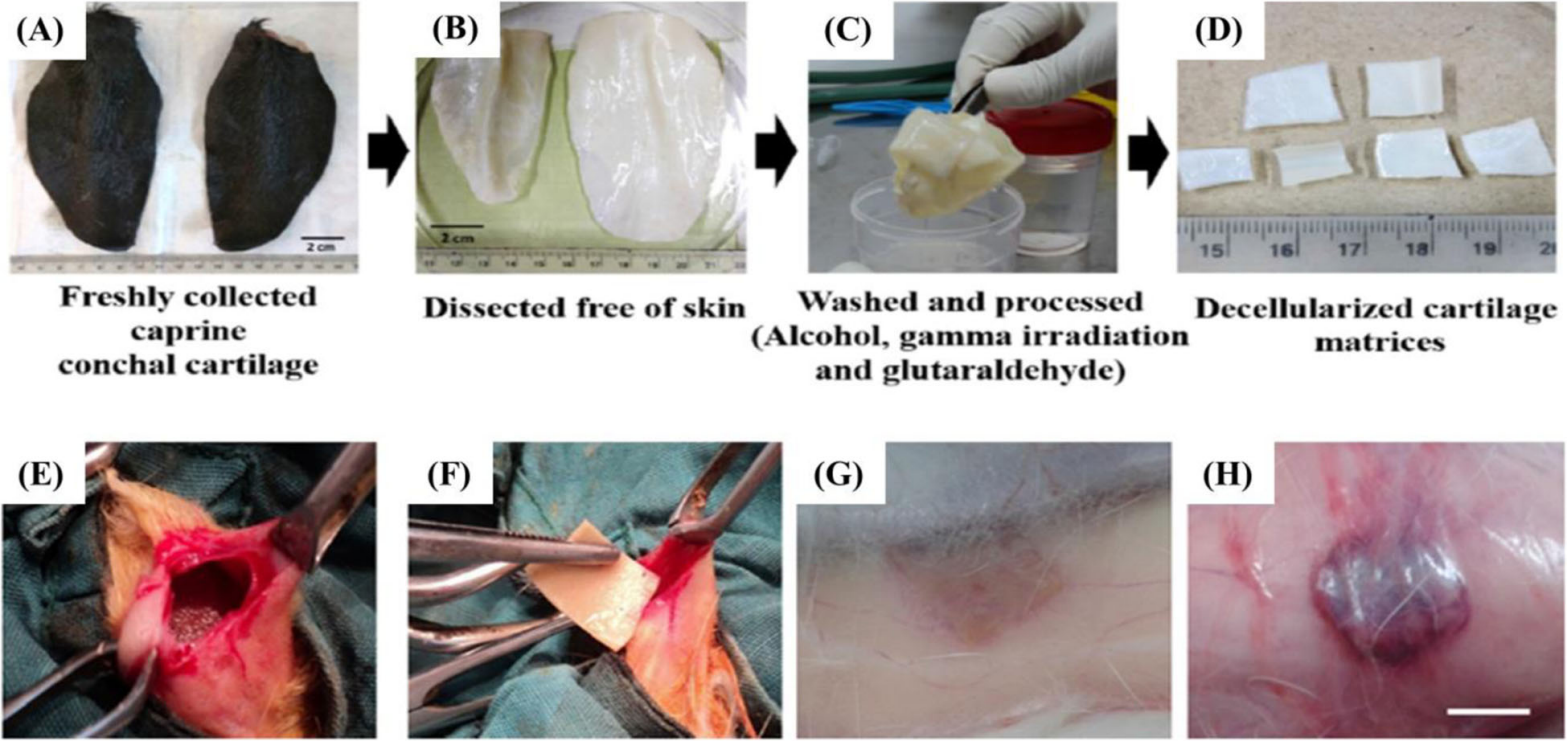
(**a**-**d**) Schematics of harvesting, processing, and decellularization of conchal cartilage. (**e** and **f**) In vivo xenoimplantation of cartilages. (**g**) Three months after the xenoimplantation, no sign of inflammation and tissue necrosis. (**h**) Native or untreated cartilage, showed necrosis of host tissue. Republished with permission of ref. [131], Das P, Singh YPP, Joardar SN, Biswas BK, Bhattacharya R, Nandi SK, et al. Decellularized Caprine Conchal Cartilage towards Repair and Regeneration of Damaged Cartilage. ACS Applied Bio Materials. 2019, Copyright (2019)

In a recent work by Piyali Das et al., decellularized caprine conchal cartilage (DC) has been used as a non-toxic and durable matrix [131]. In vivo experiments showed that DCs were well organized after the transplant, and no significant infiltration of plasma cells, immature fibroblasts, lymphocytes, and macrophage was observed (Fig. 13). Therefore, according to studies, these xenocompatible matrices are usable in the regeneration of musculoskeletal systems, especially cartilage tissues.

In addition to the biological materials discussed above, many materials have been inspired by nature **(inspired materials**) to be used in tissue engineering and regenerative medicine. A good example is marine mussels, which by secreting mussel adhesive proteins (MAPs) can adhere to different surfaces [132, 133]. Among the six *Mytilus edulis* foot proteins (Mefps) of MAPs known to be Mefp-1, Mefp-2, Mefp-3, Mefp-4, Mefp-5 and Mefp-6, components of Mefp-3, Mefp-5 and Mefp- 6 have the most critical role in adhesion [134–136]. Since the last three listed contain 3,4-dihydroxyphenylalanine (DOPA), the researchers concluded that DOPA is a significant factor in the interaction between materials and surfaces [137]. Also, since catechol groups present in the molecule can adhere to wet surfaces in the environment, especially in biological systems, researchers have done extensive research on them [138, 139]. According to the aforementioned, hydrogels prepared from functionalized materials with catechol groups have been used in tissue engineering, in particular, musculoskeletal tissue engineering. For example, Zhang et al. used a hydrogel/ fiber scaffold made of alginate, which was functionalized with DOPA and created alginate-DOPA beads [140]. Finally, they observed increased viability, cell proliferation, and osteogenic differentiation of stem cells in the alginate-DOPA hydrogel. Another inspired substance is mussel-inspired poly norepinephrine (pNE), which acts as a transmitter and catecholamine hormone in the human brain [141]. Ying Liu et al. prepared polycaprolactone (PCL) fibers with the appropriate diameter and then coated the surface with pNE [142]. They did this to integrate the regenerated muscle layer into the surrounding tissues and simulate mechanical strength to native tissue in the affected area. Finally, they achieved promising results with pNE-modified PCL fibers for use in muscle tissue engineering.

### Synthetic polymers for musculoskeletal and cartilage tissue engineering

Unlike biological polymers, synthetic polymers can easily be manipulated, depending on the needs [143]. Therefore, in musculoskeletal tissue engineering, depending on the type of tissue, for example, bone, cartilage, muscle, ligament and tendon, scaffolds with different mechanical strengths and different degradation rates can be constructed using synthetic polymers. These polymers have disadvantages, including poor biological properties and poor biocompatibility due to the degradation and release of substances such as acidic products [144]. Due to the wide variation in the properties of various tissues, it is not possible to create the required physical and chemical properties in the scaffold using only natural materials or synthetic polymers. Therefore, in tissue engineering, it is preferred that composites, or hybrid materials, such as polymer-polymer blends, polymer–ceramic blends and co-polymers, be used.

For example, the bone tissue, in addition to organic materials (collagen), contains inorganic components such as calcium phosphate (CaP) minerals. A primary CaP mineral of bone is Hydroxyapatite (HAP) (Ca_10_(PO_4_)_6_(OH)_2)_. So, incorporation of HAP in polymeric matrices can promote the response of bone cells [82]. In recent years, biomimetic mineralized scaffolds have been more considered due to their suitable chemical, physical, and biological properties for the engineering of hard tissues. HAP has been widely studied in biomedical applications due to its bioactivity, biocompatibility, and osteoconductivity. Previous studies demonstrated that nano-HAP could enhance the adhesion and proliferation of osteoblasts. It seems that composite scaffolds based on nano-HAP and natural or synthetic biomaterials can be more suitable for bone regeneration [83].

Therefore, the blending of minerals as inorganic bioactive materials with polymers can support cell attachment, proliferation, and differentiation in bone tissue. Chetna Dhand et al. have fabricated a composite scaffold using collagen nanofibers combined with catecholamines and CaCl_2_ [145]. In this study, divalent cation led to oxidative polymerization of catecholamines and crosslinking of collagen nanofibers. The introduction of divalent cation and mineralization of the scaffold by ammonium carbonate caused the prepared structure to have better mechanical properties. In vitro studies have also shown that scaffolds support the expression of osteogenic markers such as osteocalcin, osteopontin, and bone matrix protein [145]. Most of the synthetic polymers used in musculoskeletal tissue engineering, alone or in combination with natural biomaterials, include poly ε-caprolactone (PCL), polyurethane (PU), polylactic acid (PLA), polyglycolic acid (PGA), polyphosphazene and poly (propylene fumarates) [146–149]. Poly caprolactone, as an FDA approved polymer, because of relatively low melting point (55–60 °C) and excellent blend-compatible with different additives, can be used for fabrication of various scaffolds with specific shape [63]. Despite the mentioned advantages, PCL has some drawbacks, for example, in vivo degradation rate that is slow, and lack of bioactivity that limits its application in bone tissue engineering. The combination of PCL with other biomaterials such as silica, β-tricalcium phosphate, and hydroxyapatite can overcome these limitations. PCL composite nanofibers containing nHA enhance elastic modulus, cellular adhesion and proliferation, and osteogenic differentiation [150]. Also, PCL nanofibers are extensively employed in tendon tissue engineering. PCL has a hydrophobic and semi-crystalline structure that leads to its low degradation rate so that it can be used as a scaffold in the healing process of damaged tendons [9, 151]. But, the hydrophobic nature of PCL leads to insufficient cell attachment, poor tissue integration, and little wettability in tissue engineering [152]. GuangYang et al. fabricated composite scaffolds based on electrospun PCL and methacrylated gelatin (mGLT) [9]. They used a photocrosslinking method for preparation of multilayered scaffold, which mimics the native tendon tissue [9].

Another suitable synthetic polymer for musculoskeletal tissue engineering is polyurethane (PU). Polyurethanes (PUs), as elastic polymers, due to their features such as mechanical flexibility, biocompatibility, biodegradability, and tunable chemical structures have been considered in regeneration of cartilage, bone and soft tissue [96]. Also, PU due to its soft tissue-like properties and electroactivity can be employed as a scaffold in muscle tissue engineering [153]. Previous studies demonstrated electroactive polymers could support cell proliferation and differentiation [154].

**Fig. 14:**
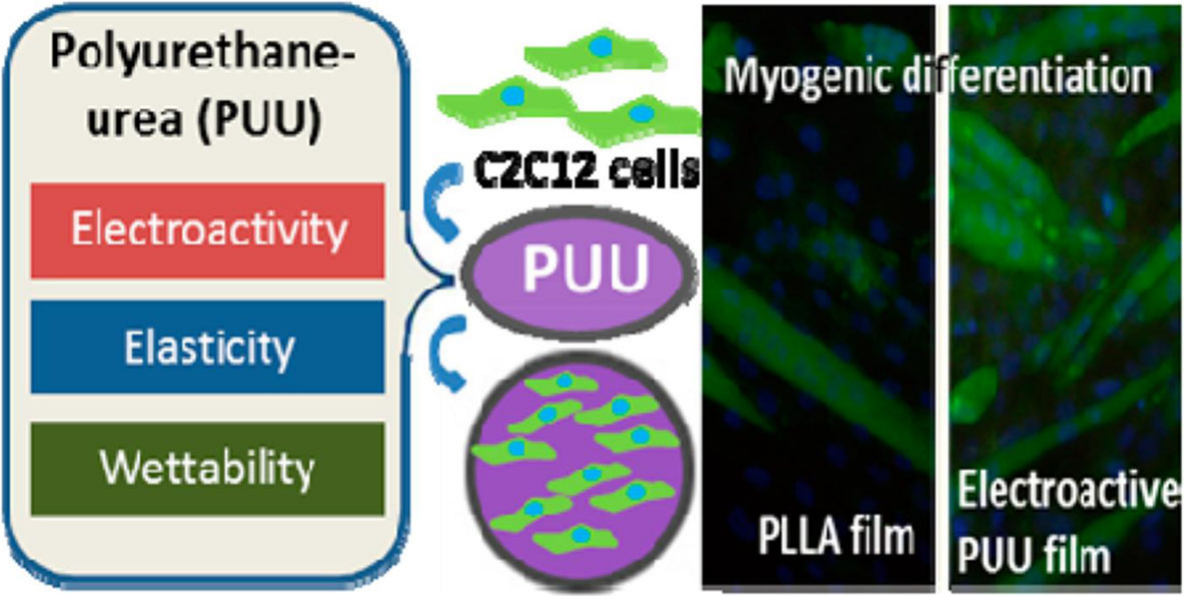
Electroactive Polyurethane-Urea elastomers with tunable hydrophilicity for skeletal muscle tissue engineering. Reprinted with permission from ref. [153], Chen J, Dong R, Ge J, Guo B, Ma PX. Biocompatible, biodegradable, and electroactive polyurethane-urea elastomers with tunable hydrophilicity for skeletal muscle tissue engineering. ACS applied materials & interfaces. 2015;7(51):28273–85, Copyright (2019)

Jing Chen et al. designed an electroactive scaffold based on polyurethane-urea (PUU) co-polymers with elastomeric properties and amine capped aniline trimer (ACAT), as an illustrative component of skeletal muscle regeneration, using C2C12 myoblast cells [153]. Also, for improving surface hydrophilicity of co-polymers, dimethylol propionic acid (DMPA) was used (Fig. 14). Results indicated that the PUU co-polymer scaffolds were not cytotoxic and improved the adhesion and proliferation of C2C12 myoblast cells. Also, C2C12 myogenic differentiation studies were investigated by analyzing myogenin (MyoG) and troponin T1 genes. The results showed the expression of these genes in electroactive PUU co-polymer groups were significantly higher than other groups [153].

PU can deposit CaPs on their surface that lead to promoting osteoconductivity. Meskinfam et al. fabricated bio-mineralized PU foams based on calcium and phosphate ions. They showed that bio-mineralization plays a vital role in improving the mechanical properties of scaffolds. It is also said that through this, an appropriate surface for cell attachment and proliferation can be provided [155].

Polyglycolic and polylactic acid, as polyester polymers, are widely used in tissue engineering because of their biodegradability and biocompatibility. Polyesters as mentioned above, have also been used to repair various tissues of the musculoskeletal system, including cartilage, bone, tendon, ligament, meniscus, muscle, bone–cartilage interfaces and bone–tendon interfaces [156–158]. Also, polyphosphazene as biodegradable inorganic polymers have vast potential for using in tissue engineering [159]. Polyphosphazenes are subjected to hydrolytic degradation, and the derived products from their degradation are not toxic [160]. So, These have been widely used in drug delivery and tissue engineering, in particular, musculoskeletal tissue engineering, due to their non-toxic degradation products, hydrolytic instability, matrix permeability, and ease of fabrication [159–161]. A study has shown that this polymer increases adhesion and proliferation of osteoblasts [162]. In addition to bone healing, polyphosphazene has proven to be very good in restoring and repairing other musculoskeletal tissue, such as the tendon and ligament [163]. Along with the mentioned polymers, poly (propylene fumarate) is another case of polymers used in musculoskeletal tissue engineering for cartilage, bone, tendon, and ligament [164–168].

**Fig. 15:**
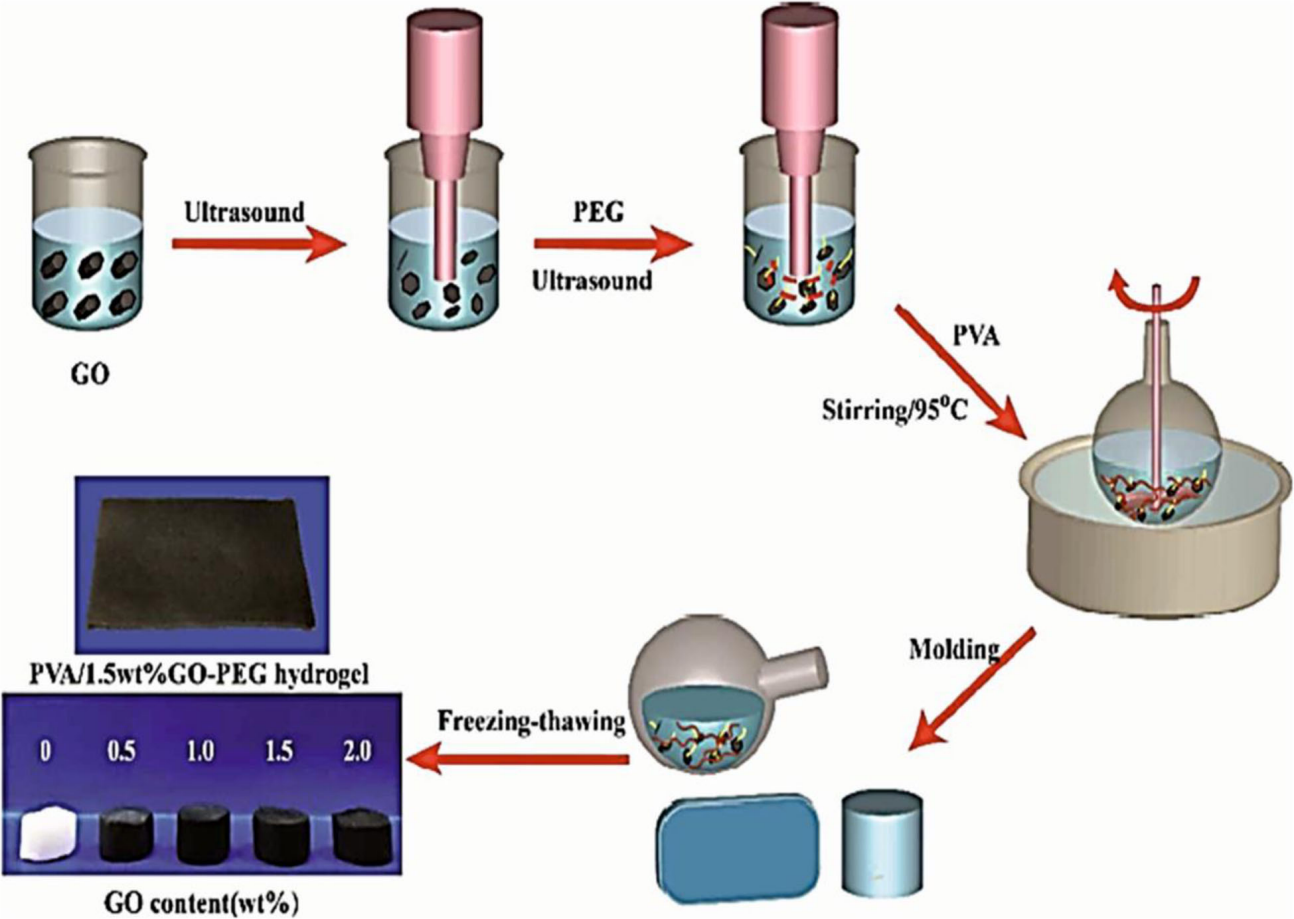
Preparation of PVA/GO-PEG nano-composite by the freezing-thawing method. Reprinted with permission from ref. [172], Meng, Y., et al., In situ cross-linking of poly (vinyl alcohol)/graphene oxide–polyethylene glycol nanocomposite hydrogels as artificial cartilage replacement: intercalation structure, unconfined compressive behavior, and biotribological behaviors. The Journal of Physical Chemistry C, 2018. 122(5): p. 3157–3167, Copyright (2019)

Among the synthetic polymers, poly (ethylene glycol) (PEG), polyglycolic acid (PGA), poly-L-lactic acid (PLLA), polyurethane (PU) and PGA-PLLA copolymers are widely used in cartilage tissue engineering because of their effectiveness as scaffolds for chondrocyte delivery [169]. In particular, poly (ethylene glycol) (PEG) is widely used as a polyether in cartilage tissue engineering. To improve the mechanical properties of the PEG, including the strength and compression modulus, it can be combined with various natural and synthetic materials [170, 171]. Yeqiao Meng et al. fabricated nanocomposite hydrogel based on Poly(vinyl alcohol) (PVA), graphene oxide (GO) and polyethylene glycol (PEG) as an artificial cartilage replacement with the name of PVA/GO-PEG by freezing/thawing method (Fig. 15) [172]. They found that synthetic nano-composite has improved mechanical properties and excellent lubrication.

## Conclusions

The occurrence of musculoskeletal injuries or diseases and subsequent functional disorders are one of the most difficult challenges in human health care. Tissue engineering is a new and promising strategy in this regard that introduces biomaterials as extracellular-mimicking matrices for controlling cellular behaviors and subsequent regeneration of damaged tissues. Different types of natural and non-natural biomaterials have been developed for use in musculoskeletal tissue engineering. Depending on the nature of the target tissue and their mechanical, chemical, and biological properties, different biomaterials can be used either singly or in combination, or with other additive materials.

## Data Availability

Not applicable.

## Abbreviations

3D: 3-Dimensional
ACAT: amine capped aniline trimer
ACs: Articular Chondrocytes
ACTN2: Alpha actinin skeletal muscle 2
ALP: Alkaline phosphatase
BG: Bioactive Glass
DMPA: dimethylol propionic acid
DOPA: 3,4-dihydroxyphenylalanine
ECM: Extracellular Matrix
GAGs: Glycosaminoglycans
GC: Glass-Ceramics
GelMA: Gelatin Methacrylate
GO: Graphene oxide
HA: Hyaluronic acid
HWJMSCs: Human Wharton’s Jelly Mesenchymal Stem Cells
M/T/L: Meniscus/Tendon/Ligament
MAPs: Mussel adhesive proteins
Mefps: *Mytilus edulis* foot proteins
Mkx: Mohawk homeobox
MSCs: Mesenchymal stem cells
MWNTs: Multiwall Carbon Nanotubes
MyoG: Myogenin
nHAP: Nano hydroxyapatite
Ocn: Osteocalcin
Opn: Osteopontin
PEG: Polyethylene glycol
PGA: Poly (glycolic acid)
PLA: Poly (lactic acid)
pNE: norepinephrine
PUU: Polyurethane-urea
PVA: Poly(vinyl alcohol)
RGD: Arginine, Glycine, and Aspartate
Runx2: Runt-related transcription factor 2
SA: Sodium Alginate
SCX: Scleraxis
SF: Silk fibroin
SOX 9: SRY-box 9
TNMD: Tenomodulin
VML: Volumetric Muscle Loss
